# Denoising Algorithm for CFA Image Sensors Considering Inter-Channel Correlation

**DOI:** 10.3390/s17061236

**Published:** 2017-05-28

**Authors:** Min Seok Lee, Sang Wook Park, Moon Gi Kang

**Affiliations:** 1 School of Electrical and Electronics Engineering, Yonsei University, 50 Yonsei-Ro, Seodaemun-Gu, Seoul 03722, Korea; gasinamul@empal.com; 2 Medical Device Development Centre, Daegu-Gyeongbuk Medical Innovation Foundation, 80 Cheombok-Ro, Dong-gu, Daegu 41061, Korea; scrambls@hanmail.net

**Keywords:** color filter array image sensor, spatial-temporal filter, video denoising, inter-channel correlation

## Abstract

In this paper, a spatio-spectral-temporal filter considering an inter-channel correlation is proposed for the denoising of a color filter array (CFA) sequence acquired by CCD/CMOS image sensors. Owing to the alternating under-sampled grid of the CFA pattern, the inter-channel correlation must be considered in the direct denoising process. The proposed filter is applied in the spatial, spectral, and temporal domain, considering the spatio-tempo-spectral correlation. First, nonlocal means (NLM) spatial filtering with patch-based difference (PBD) refinement is performed by considering both the intra-channel correlation and inter-channel correlation to overcome the spatial resolution degradation occurring with the alternating under-sampled pattern. Second, a motion-compensated temporal filter that employs inter-channel correlated motion estimation and compensation is proposed to remove the noise in the temporal domain. Then, a motion adaptive detection value controls the ratio of the spatial filter and the temporal filter. The denoised CFA sequence can thus be obtained without motion artifacts. Experimental results for both simulated and real CFA sequences are presented with visual and numerical comparisons to several state-of-the-art denoising methods combined with a demosaicing method. Experimental results confirmed that the proposed frameworks outperformed the other techniques in terms of the objective criteria and subjective visual perception in CFA sequences.

## 1. Introduction

Image denoising is an indispensable part of digital image processing on account of the continuous need for high-quality images. When images are obtained by a sensor system, noise inevitably arises in the images. Noise can be amplified by electronic sensor gains obtained by any post-image processing that increases the intensity range. Therefore, noise suppression must be conducted in advance. A denoising algorithm is necessary to improve image quality as well as to provide rectified input images that are additionally processed in tasks, such as segmentation, feature extraction, and texture analysis.

Many denoising filters have been presented in the literature. In the spatial domain, the use of patch-based approaches has become very popular in recent years. The nonlocal means (NLM) filter is one of the most renowned denoising methods [1]. As an averaging-based filter, which directly smooths pixel values in the spatial domain, the NLM filter is an effective denoising method. When using the NLM filter, the similarity between the center patch and neighboring patches can be utilized for weight calculation. The nonlocal property of the images (self-similarity) has been adopted for denoising methods based on patch-based difference (PBD) refinement [2]. Hence, an improved patch-based denoising method, block-matching, and a 3D filtering (BM3D) algorithm were presented in Ref. [3].

To restore degraded image sequence data, denoising methods extended to the temporal domain have also been presented in recent years [4,5,6,7,8,9,10,11]. In the denoising process for monochromatic data, explicit motion representation refers to the detection of the true motion trajectories of a physical point via motion estimation and compensation. Many temporal filters, such as the locally adaptive linear minimum-mean-squared-error spatio-temporal filter (3D-LLMMSE) [4] and the multi-hypothesis motion-compensated filter (MHMCF) [5,6], adopt this approach. The motion-compensated denoising algorithm based on 3D spatiotemporal volume for random and fixed-pattern noises [10] and using regularized optical flow [11] were presented recently. This motion-compensated filtering approach can be effective in preventing motion blurring during video denoising processes.

**Figure 1:**
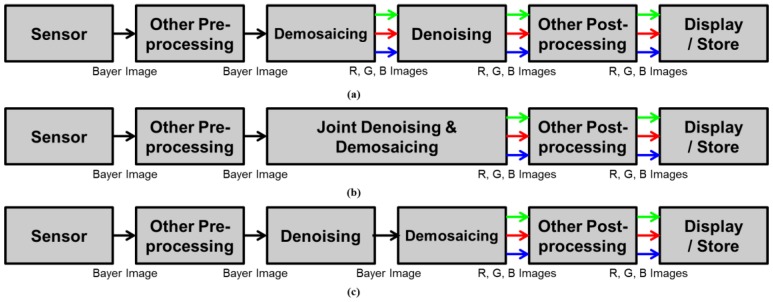
Image Processing Pipelines; (**a**) demosaicing, and then denoising; (**b**) joint denoising and demosaicing; (**c**) the proposed framework (denoising, and then demosaicing).

Furthermore, the denoising algorithm can be used with a color filter array (CFA) pattern, which is found in most cameras that use single sensors to obtain color images. The CFA pattern usually has three alternating color channels. Because each color channel shares a sensor plane at the sacrifice of the under-sampled spatial resolution in each channel, demosaicing must be performed to obtain a full resolution image in each channel [12,13,14,15,16,17]. The most renowned CFA is the Bayer pattern [18], which consists of a green channel of half the number of whole pixels for the red and blue channels of the quarter, respectively. Recently, several other CFA patterns have been developed with the objective of enhancing sensitivity [19,20,21,22]. In addition, demosaicing methods for various CFA patterns have been presented recently [23,24]. The denoising filter is typically applied separately after demosaicing for the three color channels, as shown in Figure 1a. In this case, there is no need to consider the under-sampled resolution of each channel. However, demosaicing changes the statistical characteristics of the noise and introduces spatial correlations [25]. Moreover, demosaicing relies on chromatic aberration, which influences the spatial and spectral correlation through artifacts such as blur and mis-registration [26]. Thus, the noise spreads spatially, which makes the denoising task after demosaicing more complicated. 

As a unified estimation procedure, denoising and demosaicing, are conducted simultaneously in [27,28,29], as shown in Figure 1b. These approaches try to obtain more efficient and cost-efficient solutions by jointly considering denoising and demosaicing. The total least square based filter is adopted to remove both signal-dependent and signal-independent noise factors and conduct demosaicing in [27]. Denoisaicing, which is a complex word for denoising and demosaicing, is presented in [28]. In [29], the total variation minimization is utilized to suppress sensor noise and demosaicing error.

Moreover, denoising for the three interpolated channels incurs high computational costs. To address these disadvantages, some denoising methods have been directly used with the CFA [30,31,32], as shown in Figure 1c. In the proposed approach, a denoising algorithm is also performed in the CFA pattern before the demosaicing process, as shown in Figure 1c. By employing these typical structures, many demosaicing methods have been developed for Bayer pattern [12,13,14,15,16]. Most demosaicing algorithms are based on the assumption that the differences or ratios among the channels are smoother than the intensities of the channels, which is known as the inter-channel correlation [12,13,14,15]. In other words, the high frequencies of the red, green, and blue channels are assumed to be similar. The high frequencies among the different channels can be jointly exploited during an interpolation procedure to overcome the degraded spatial resolution caused by under-sampling. Most demosaicing algorithms consider the inter-channel correlation to suppress color artifacts and interpolation errors.

As in demosaicing, consideration of the inter-channel correlation is key to performing the denoising process in the CFA sequence. In the proposed method, the spatio-spectral-temporal correlation is considered for CFA denoising. The main contribution of this paper is twofold. First, a spatial filter considering spatio-spectral correlation is proposed to effectively remove noise while preserving edge details. The clarity along the edges and details is required for denoising in the spatial domain. However, edge estimation on an alternating under-sampled grid can be very difficult. Hence, the spatio-spectral correlation must be considered in the spatial filter, and the inter-channel correlation must be analyzed to detect the edges and details in the under-sampled pattern. Owing to the alternating under-sampling of the CFA pattern, the inter-channel correlation is difficult to consider in the direct denoising process. Hence, when conventional methods are used for the CFA pattern, spatial resolution degradation occurs. Conventional methods, which involve direct removal of noise from CFA, do not take into account the inter-channel correlation. To overcome the spatial resolution degradation while removing noise, both inter-channel and intra-channel correlations are used in the direct denoising process. In the proposed spatial filter, the patch is computed from the pixel values of all the color channels, such as a monochromatic NLM filter. The PBD values of the pixel locations are obtained from the intra-channels. Then, PBD refinement is conducted by considering both the intra-channel and inter-channel correlations in the CFA pattern. 

Second, a motion-compensated temporal filter is proposed that removes the noise in the temporal domain without motion artifacts and considers the tempo-spectral correlation. Most existing denoising algorithms that consider motion information have been proposed for monochromatic sequence data and are therefore difficult to apply directly in the CFA sequence. Owing to the alternating under-sampling of the CFA pattern, motion estimation and compensation are difficult to perform in temporal filtering. In the proposed temporal filter, the inter-channel correlation is considered in the motion estimation and compensation processes. To find the motion trajectory in a CFA sequence, a candidate motion vector is estimated from the CFA pattern by using hypothetical intensity maps. To consider the inter-channel correlation, these proposed hypothetical intensity maps are formed by pixels neighboring the center pixel. After determining the motion trajectories from the candidate motion vectors, the proposed motion-compensated temporal filter is directly performed on the CFA sequence. A motion adaptive spatio-spectral-temporal filter is proposed to eliminate motion blurring while maintaining the advantages of the temporal filter. It consists of a filter that operates in both the spatial and temporal domains, and the filtered results of these domains are combined using a motion adaptive value. The motion adaptive detection value is estimated by the variance of the absolute difference calculated from the CFA sequence. It adaptively controls the ratio of the results of the spatial and temporal filters. Then, the denoised CFA sequence without motion blurring is acquired.

The remainder of this paper is organized as follows: Section 2 provides the background of the proposed method. In addition, we explain the proposed algorithm for use with the CFA sequence. The motivation and novelty of the proposed method are described in Section 3. In Section 4, the experimental results are given, including comparisons with conventional methods for test images with varying noise levels. We conclude the paper in Section 5.

## 2. Background

### 2.1. Noisy CFA Sequence

Using the noise model described in this paper, a true pixel value (noisy free pixel value), $x_{m,n,t}$, is obtained in the presence of an additive zero-mean white independent and identically distributed (i.i.d.) Gaussian noise value, $v_{m,n,t}$, with a standard deviation of σ. The measured noisy pixel value, $y_{m,n,t}$, can be represented as:

(1) $$y_{m,n,t}=x_{m,n,t}+v_{m,n,t}.$$

**Figure 2:**
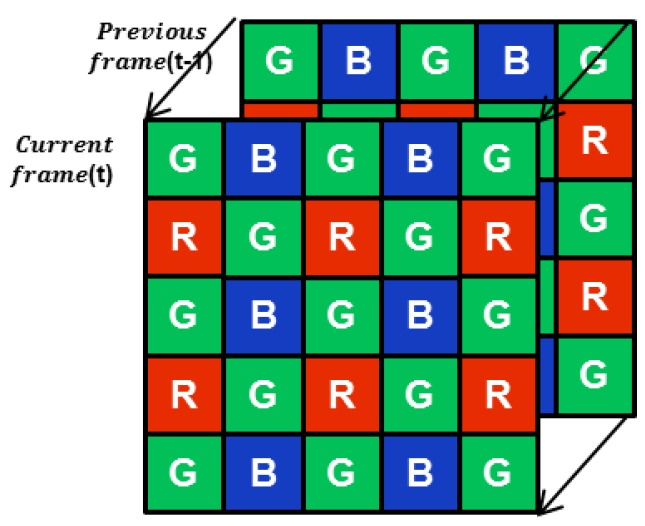
CFA Sequence to be denoised.

In the CFA sensor, the noisy measurements in support of the same channel are two pixels apart, as shown in the Figure 2. Thus, the neighbors for the image denoising must be collected from the larger region compared to monochromatic image denoising, which results in artifacts of the over-smoothness and resolution degradation in the denoised image. The effective exploitation of the nearest neighbors of the different channels based on the inter-channel correlation is the key to the denoising problem of the CFA sensor denoising.

### 2.2. Overall Denoising Procedure

**Figure 3:**
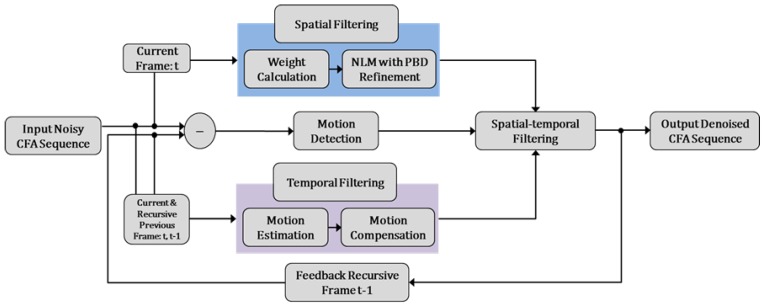
Block diagram of the proposed denoising algorithm.

The overall block diagram of the proposed algorithm is shown in Figure 3. First, the motion adaptive detection value is acquired between a noisy current frame and a denoised previous frame. Then, the respective results of spatial filtering and temporal filtering are combined into spatio-spectral-temporal filtering by using a motion adaptive value. The proposed spatial filter and temporal filter are separately performed in the respective spatial and temporal domains. The proposed filter is based on the inter-channel correlation of the CFA sensor image. Finally, the denoised current frame is stored in the frame memory and fed back to the next denoising process.

## 3. Proposed Method

### 3.1. Inter-Channel Correlated Spatial Filter

#### 3.1.1. NLM Filter with PBD Refinement for the CFA Image

**Figure 4:**
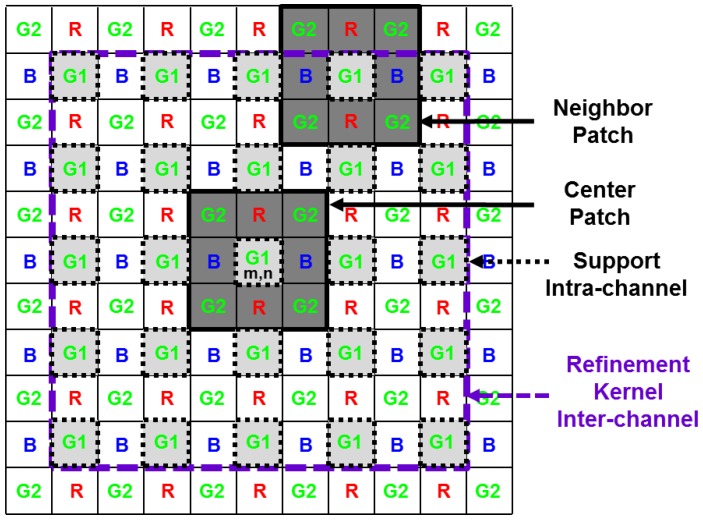
Boundaries used in the proposed method.

The boundary terms used in the proposed method are illustrated in Figure 4 and defined as follows. The center patch is the rectangular windowed neighborhood centered on a denoising pixel, and the neighbor patch is the rectangular windowed neighborhood centered on a neighboring pixel within the support for the center location (m,n). The support represents the set of intra-channel pixels belonging to the same channel centered on the pixels in the whole refinement kernel, including the center location (m,n). The refinement kernel is the rectangular windowed inter-channel neighborhood belonging to all channels centered on the location (m,n).

The modified equation of the NLM filter [1] as the spatial filter performed in the CFA image is mainly addressed as follows:

(2) $${\hat{x}}_{m,n,t}^{sf}=\frac{\sum_{i=(m-S)/2}^{(m+S)/2}\sum_{j=(n-S)/2}^{(n+S)/2}w_{m,n,2i,2j}y_{2i,2j,t}}{\sum_{i=(m-S)/2}^{(m+S)/2}\sum_{j=(n-S)/2}^{(n+S)/2}w_{m,n,2i,2j}},$$

where ${\hat{x}}_{m,n,t}^{sf}$ is the estimated pixel value obtained by the NLM spatial filter for the CFA image. Owing to the mosaic pattern, the pixel location to be filtered (i,j) is multiplied by two because each pixel position within the same color channel is filtered by the same kernel after every two pixels along the horizontal and vertical directions, as shown in Figure 4. In addition, S represents half the size of the support and is set as an even number. The optimal support size is 25×25, as explained in Equation (2), which is large for a conventional NLM denoising filter. Nevertheless, the neighboring pixels for denoising have to be gathered from pixels that are two unit positions away, both the horizontal and vertical directions because of the alternating under-sampled grid. Thus, only half of the pixels in the support contributed to denoising. The larger the support size, the smoother is the denoising result, as addressed in [1]. Instead of enlarging the support size, utilizing the PBD images from the inter-channel locations including the nearest neighbors induced the pixels in different channels to participate in denoising. Thus, the PBD refinement method based on the inter-channel correlation prevents denoising from over-smoothness and preserves more details.

The equation of the proposed spatial filter is based on the NLM filter with PBD refinement [2] for the CFA image. It is defined as:

(3) $${\hat{x}}_{m,n,t}^{sf}=\frac{\sum_{i=(m-S)/2}^{(m+2)/2}\sum_{j=(n-2)/2}^{(n+2)/2}(h-{\bar{D}}_{m,n,2i,2j})y_{2i,2j,t}}{\sum_{i=(m-S)/2}^{(m+2)/2}\sum_{j=(n-2)/2}^{(n+2)/2}\left(h-{\bar{D}}_{m,n,2i,2j}\right)}.$$

Then, the corresponding weight $w_{m,n,2i,2j}$ is given by:

(4) $$w_{m,n,2i,2j}=\{\begin{array}{cc} h-{\bar{D}}_{m,n,2i,2j} & ,{\bar{D}}_{m,n,2i,2j}<h\\ 0 & ,otherwise \end{array},$$

where h represents a smoothing threshold, and ${\bar{D}}_{m,n,2i,2j}$ is refined by the PBD values. It is represented as:

(5) $${\bar{D}}_{m,n,2i,2j}=\sum_{q=-S}^{S}\sum_{r=-S}^{S}a_{q,r}D_{m+q,n+r,2i+q,2j+r},$$

where the PBD value $D_{m,n,2i,2j}$ is computed as:

(6) $$D_{m,n,2i,2j}=\frac{\sum_{k=-S}^{S}\sum_{l=-S}^{S}\left|y_{2i+k,2j+l,t}-y_{m+k,n+l,t}\right|}{N_{P}}.$$

The PBD value $D_{m,n,2i,2j}$ is acquired by subtracting the center patch and the neighboring patches, as shown in Figure 3, and the $D_{m,n}$ PBD image:

(7) $$D_{m,n}={\left[D_{m,n,m-S,n-S},\ldots ,D_{m,n,m,n},\ldots ,D_{m,n,m+S,n+S}\right]}^{T},$$

where $D_{m,n}$ is a lexicographically ordered sub-image within the same support region, such as in Equation (3). In addition, P represents half the size of the patch centered on the pixel locations (2i,2j) and (m,n). $N_{P}$ represents the number of pixels in the patch range. The threshold value, h, of the proposed NLM filter in Equation (3) has a smoothing effect and bandwidth to enable execution of the kernel method. 

#### 3.1.2. Inter-channel Correlated PBD Image Calculation in the Refinement Kernel

**Figure 5:**
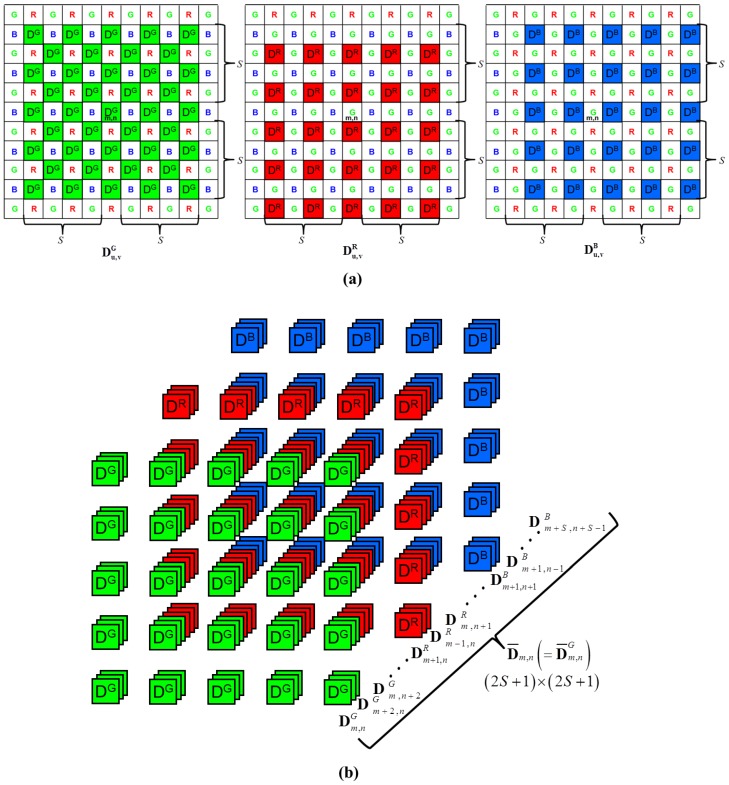
Schematic representation of the inter-channel correlated PBD refinement procedure. (**a**) the PBD image calculation procedure; (**b**) the PBD images stacking and refinement procedure.

In this paper, we examine the problem of inter-channel correlated denoising to overcome the spatial resolution degradation of the alternating under-sampled CFA image. Thus, the proposed PBD refinement is performed with consideration of the inter-channel correlations. The PBD value $D_{m,n,2i,2j}$ is expressed as a lexicographically ordered group of the PBD image $D_{m,n}$ in the same way as in Equation (7) and within the same support region as in Equation (3). As shown in Figure 5a, $D_{u,v}^{G}$ denotes the PBD image with the support region centered on the location (u,v) of the G1 and G2 channel positions, where (u,v)∈{(m−S,n−S),⋯,(m,n−2),(m,n),(m,n+2),⋯,(m+S,n+S)}∪{(m−S+1,n−S+1),⋯,(m+1,n−1),(m+1,n+1),⋯,(m+S−1,n+S−1)}.

Similarly, $D_{u,v}^{R}$, and $D_{u,v}^{B}$ denote the PBD images with the support regions centered on the location (u,v) corresponding to the R and B channels within the refinement kernel, respectively. As a schematic representation of Equation (5), Figure 5b shows the inter-channel correlated PBD refinement procedure, which means that the refined PBD image ${\bar{D}}_{m,n}$ is updated not only from the PBD images of the same channel at the (m,n) location (the G1 channel for the example shown in Figure 5b), but also from the PBD images of the other channels (the R and B channels for the example shown in Figure 5b). In other words, the PBD images at all the locations within the S range of the refinement kernel are calculated and stacked for all the channels, such as in Figure 5b. Then, the weighted average for the PBD images is conducted by using Equation (5). With this inter-channel correlated PBD image calculation, the proposed NLM-based spatial filter can reflect the inter-channel information in the denoising process. 

#### 3.1.3. Weight Calculation in the Refinement Kernel

As shown in Figure 5, the PBD images in the kernel $D_{u,v}^{G}$, $D_{u,v}^{R}$, and $D_{u,v}^{B}$ at the G channel location are refined by using the weight $a_{q,r}$ in Equation (5), which is computed as:

(8) $$a_{q,r}=\frac{b_{q,r}}{\sum_{q=-S}^{S}\sum_{r=-S}^{S}b_{q,r}},0\leq a_{q,r}\leq 1,$$

(9) $$b_{q,r}=\{\begin{array}{cc} 1-k\frac{\sum_{i=(m-S)/2}^{(m+S)/2}\sum_{j=(n-S)/2}^{(n+S)/2}\left|D_{m+q,n+r,2i+q,2j+r}-D_{m,n,2i,2j}\right|}{N_{S}}, & q+r\;is\;even\;number(G\;channel\;localtion),\\ 1-s_{q,r}k\frac{\sum_{i=(m-S)/2}^{(m+S)/2}\sum_{j=(n-S)/2}^{(n+S)/2}\left|D_{m+q,n+r,2i+q,2j+r}-D_{m,n,2i,2j}\right|}{N_{S}}, & otherwise(R,B\;channel\;location), \end{array}$$

where $N_{S}=(2S+1)(2S+1)$ is the number of pixels in the refinement kernel. k is a scalar, and we set k=0.1 as a predefined constant value. When q and r are even numbers, $D_{m+q,n+r,2i+q,2j+r}$ is at the same G channel location as $D_{m,n,2i,2j}$. Moreover, $D_{m+q,n+r,2i+q,2j+r}$ and $D_{m,n,2i,2j}$ are in the G1 and G2 channels, respectively. In this case, both q and r are odd numbers. When the sum of q and r is an even number, $D_{m+q,n+r,2i+q,2j+r}$ and $D_{m,n,2i,2j}$ are all in intra-channel locations. 

The proposed PBD refinement method is performed differently by the color saturation value $s_{q,r}$ depending on the locations of the PBD values, as in Equation (3). The PBD values to be refined are all in intra-channel locations, and the weight for refinement $b_{q,r}$ is not attenuated, regardless of the color saturation value. On the other hand, when the PBD values are all in inter-channel locations, the weight for refinement $b_{q,r}$ is attenuated by the color saturation value. The weight $s_{q,r}$ is used to constrain the refinement in different ways for the respective intra-channel and inter-channel locations and with consideration of the color saturation values [33] given by:

(10) $$s_{q,r}=\frac{\sqrt{{\left(D_{q,r}^{R}-D_{q,r}^{G}\right)}^{2}+{\left(D_{q,r}^{G}-D_{q,r}^{B}\right)}^{2}+{\left(D_{q,r}^{B}-D_{q,r}^{R}\right)}^{2}}}{(D_{q,r}^{R}+D_{q,r}^{G}+D_{q,r}^{B})/3},0\leq s_{q,r}\leq 1.$$

Within the low color saturation regions, $s_{q,r}$ is close to one. Then, the refinement method is conducted considering both intra-channel and inter-channel correlations. However, within the high color saturation regions, $s_{q,r}$ become small. Then, the refinement for the inter-channel locations is reduced, and the refinement for the intra-channel correlation is carried out. At the R and B channel locations, as shown in Figure 5, the PBD images are refined by using the weight $b_{q,r}$ for refinement, which is calculated as:

(11) $$b_{q,r}=\{\begin{array}{cc} 1-k\frac{\sum_{i=(m-S)/2}^{(m+S)/2}\sum_{j=(n-S)/2}^{(n+S)/2}\left|D_{m+q,n+r,2i+q,2j+r}-D_{m,n,2i,2j}\right|}{N_{S}}, & q\;and\;r\;are\;even\;numbers(R,B\;channel\;localtion),\\ 1-s_{q,r}k\frac{\sum_{i=(m-S)/2}^{(m+S)/2}\sum_{j=(n-S)/2}^{(n+S)/2}\left|D_{m+q,n+r,2i+q,2j+r}-D_{m,n,2i,2j}\right|}{N_{S}}, & otherwise(G\;channel\;location), \end{array}$$

where $D_{m+q,n+r,2i+q,2j+r}$ and $D_{m,n,2i,2j}$ are all in intra-channel locations for the R and B channel locations if both q and r are only even numbers. The weight for the refinement calculation at the R and B channel locations is measured in the same way as in the G channel location. The weight for refinement is attenuated by the color saturation value in different ways for the respective intra-channel and inter-channel locations.

### 3.2. Motion-Compensated Temporal Filter

#### 3.2.1. Inter-Channel Correlated Motion Estimation

We propose a method for estimating motion vectors for motion-compensated temporal filtering in the CFA sequence. Motion estimation and compensation are used to efficiently remove noise from video data. However, it is very difficult to estimate the actual motion in the CFA sequence. Because the CFA pattern has an under-sampled grid, motion errors can occur when using the conventional motion estimation methods for monochromatic sequences. Thus, the inter-channel correlation must be considered to estimate the motion in CFA under-sampled sequence. The proposed method takes into account the inter-channel correlation in both the motion estimation and compensation processes. In motion-compensated temporal filtering, the first procedure is to find the motion trajectory for each pixel of the reference frame. To improve motion-compensated filtering performance, it is important to predict the exact motion trajectories in the CFA sequence. A block matching-based motion estimation method that considers the inter-channel correlation for the noisy CFA sequence is presented.

**Figure 6:**
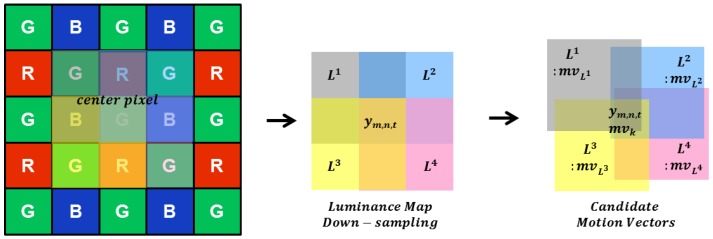
Illustrations of the motion estimation procedure.

Owing to the characteristics of the CFA sensor, applying the block matching technique to the pattern makes it difficult to predict the exact motion vector. Therefore, as illustrated in Figure 6, four hypothetical intensity maps are presented to overlap the center pixel in the search range. The intensity used in the proposed maps is pixel values of red, blue, and green color channels. These virtual maps are obtained by taking the average of the red, blue, and two green channels as pixels in four directions around the center pixel, as follows:

(12) $${L^{1}}_{m,n,t}=(y_{m-1,n-1,t}+y_{m-1,n,t}+y_{m,n,t}+y_{m,n-1,t})/4,\;{L^{2}}_{m,n,t}=(y_{m-1,n,t}+y_{m-1,n+1,t}+y_{m,n+1,t}+y_{m,n,t})/4,\;{L^{3}}_{m,n,t}=(y_{m,n,t}+y_{m,n+1,t}+y_{m+1,n+1,t}+y_{m+1,n,t})/4,\;{L^{4}}_{m,n,t}=(y_{m,n-1,t}+y_{m,n,t}+y_{m+1,n,t}+y_{m+1,n-1,t})/4,$$

where (m,n) represents the spatial location of intensity maps down-sampled from the CFA sequence within the same support region, as in Equation (3). Motion estimation based on the block matching algorithm is performed in each of the four hypothetical intensity maps. The motion trajectory at the current pixel position is estimated by extracting four candidate motion vectors from the center pixel of the CFA sequence by motion estimation.

The four candidate motion vectors in each down-sampled intensity map are calculated as:

(13) $$mv_{L^{k}}=\arg\underset{d}{\min}SAD(d;L^{k}),k=1,2,3,4,$$

where d represents a displacement vector $\left(d_{m},d_{n}\right)$ in each map, $L^{k}$ represents the four maps, and $SAD(d;L^{k})$ denotes the sum of the absolute difference, which is the block matching criterion applied to all the hypothetical maps. It is obtained by:

(14) $$SAD(d;L^{k})=\sum_{(m,n)\in S}|{L^{k}}_{m,n,t}-{L^{k}}_{m+d_{m},n+d_{n},t-1}|,k=1,2,3,4,$$

where it is calculated within search range S. In addition, $\left(m+d_{m},n+d_{n}\right)$ means the spatial displacement of each hypothetical intensity map between the current and reference frame. After obtaining the hypothetical four candidate motion vectors in the intensity map, this motion vector at the current pixel of the CFA image is up-sampled as follows:

(15) $$mv_{k}=2mv_{L_{k}},k=1,2,3,4,$$

where $mv_{k}$ represents a motion vector $\left(mv_{m}^{L_{k}},mv_{n}^{L_{k}}\right)$ in each map. Owing to the under-sampled pattern, the motion vector computed in the hypothetical map is up-sampled by two. The four candidates for the motion vector have a high inter-channel correlation at the current pixel of the CFA pattern. If the candidate motion vectors are different, the center pixel of the CFA sequence has four possible motion trajectories. On the other hand, when the candidate motion vectors are the same, the center pixel of the CFA sequence has a static motion or the same motion trajectory. These four candidate motion vectors in the CFA sequence are highly correlated with the motion state of the center pixel by using the inter-channel correlation. These estimated candidate motion vectors are used for motion-compensated temporal filtering.

#### 3.2.2. Motion-Compensated Temporal Filtering

**Figure 7:**
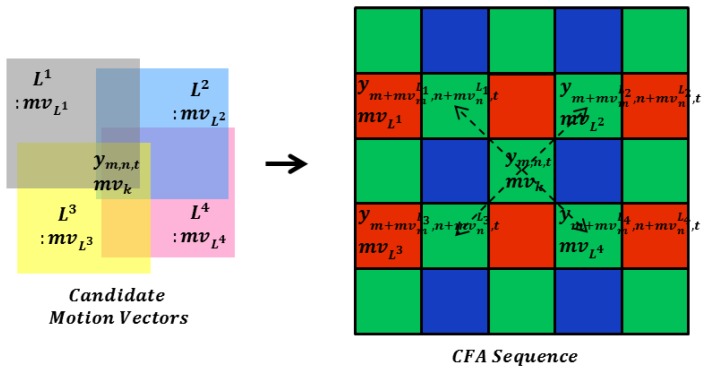
Illustrations of the motion-compensated prediction.

In motion-compensated temporal filtering, estimating accurate motion trajectories is important to prevent motion artifacts. It is difficult to find motion-compensated prediction (MCP) in the CFA sequence because of the under-sampled grid characteristics. As shown in Figure 7, four candidates of MCPs are determined by estimating the candidate motion vectors. The estimations of these four candidate motion vectors are highly correlated with center pixel’s motion trajectory. 

The proposed MCP in the CFA sequence at the spatial position (m,n) of the recursive previous frame is given by:

(16) $${\hat{x}}_{m,n,t-1}^{tf}=\sum_{k=1}^{4}\alpha_{k}y_{m,n,t-1,k}^{mc},k=1,2,3,4,$$

where $y_{m,n,t-1,k}^{mc}$ is the kth candidate of MCP. It is estimated from the pixel at the kth candidate motion vector position of the previous frame and is defined as:

(17) $$y_{m,n,t-1,k}^{mc}=y_{m+mv_{m}^{L_{k}},n+mv_{n}^{L_{k}},t-1},k=1,2,3,4.$$

Furthermore, $\alpha_{k}$ represents the weight of each MCP, as follows:

(18) $$\alpha_{k}=\frac{\sigma_{y_{m,n,t-1,k}^{mc}}^{-2}}{\sigma_{y_{m,n,t-1,1}^{mc}}^{-2}+\sigma_{y_{m,n,t-1,2}^{mc}}^{-2}+\sigma_{y_{m,n,t-1,3}^{mc}}^{-2}+\sigma_{y_{m,n,t-1,4}^{mc}}^{-2}},k=1,2,3,4,$$

where $\sigma_{y_{m,n,t-1,k}^{mc}}^{2}$ is the kth local variance of the absolute frame difference between the current pixel at spatial position (m,n) of the current frame t and four candidate motion vector positions $\left(m+mv_{m}^{L_{k}},n+mv_{n}^{L_{k}}\right)$ of the reference frame, t−1, as follows:

(19) $$\sigma_{y_{m,n,t-1,k}^{mc}}^{2}=\frac{\sum_{i=m-S/2}^{m+S/2}\sum_{j=n-S/2}^{n+S/2}{\left(|y_{m+i,n+i,t}-y_{m+i+mv_{m}^{L_{k}},n+j+mv_{n}^{L_{k}},t-1}|-\mu_{k}\right)}^{2}}{n(S)}.$$

here, $\sigma_{y_{m,n,t-1,k}^{mc}}^{2}$ is computed over the same support region S as in Equation (3), which is centered around the current pixel position (m,n) and motion vector position $\left(m+mv_{m}^{L_{k}},n+mv_{n}^{L_{k}}\right)$. Meanwhile, n(S) is the total number of pixels within the support region. The kth local mean of the absolute frame difference $\mu_{k}$ at each of the four motion vector positions $\left(m+mv_{m}^{L_{k}},n+mv_{n}^{L_{k}}\right)$ is given by:

(20) $$\mu_{k}=\frac{\sum_{i=m-S/2}^{m+S/2}\sum_{j=n-S/2}^{n+S/2}\left|y_{m+i,n+i,t}-y_{m+i+mv_{m}^{L_{k}},n+j+mv_{n}^{L_{k}},t-1}\right|}{n(S)}.$$

To minimize the temporal filtering error, the four candidates of temporal filtering results are combined by weighted averaging (16) according to the weight $\alpha_{k}$, which represents the possibility of a motion occurrence along with four candidate motion vectors in the CFA sequence. When kth $\sigma_{y_{m,n,t-1,k}^{mc}}^{2}$ is low, the possibility of a motion change along to the kth candidate motion vector is high. This means the correlation between the center pixel of the current frame and estimated pixel of the kth candidate MCP is high. In this case, more weight is given to the candidate MCP during the weighted averaging process to find MCP in the CFA sequence. On account of the weighted averaging process, the proposed MCP method not only estimates the accurate MCP, but it also achieves the denoising effect in the result of the reference frame. In the proposed motion-compensated temporal filtering, the estimated MCP in the CFA sequence considers the inter-channel correlation because it contains the different pixel channels from the center pixel position.

### 3.3. Spatio-Spectral-Temporal Filter

The overall block diagram of the proposed method is shown in Figure 3. The result of spatial filtering is based on PBD refinement. We furthermore propose motion-compensated temporal filtering in the CFA sequence based on inter-channel correlated motion estimation and compensation. In motion-compensated temporal filtering, the filtered value of the reference frame pixel is determined by MCP along the motion trajectory the result of temporal filtering. It is well known that a trade-off exists between the amount of noise removal and blurring in video denoising. To improve the noise filtering without motion blurring, motion adaptive recursive spatio-spectral-temporal filtering is presented as follows:

(21) $${\hat{x}}_{m,n,t}=\beta \times {\hat{x}}_{m,n,t}^{sf}+(1-\beta )\times {\hat{x}}_{m,n,t-1}^{tf},$$

where β represents the motion adaptive detection value as the spatio-spectral-temporal filtering coefficient between the center pixel of spatially filtered value ${\hat{x}}_{m,n,t}^{sf}$ and the motion-compensated temporal filtered value of reference frame ${\hat{x}}_{m,n,t-1}^{tf}$. This motion adaptive detection value is optimally calculated by linear minimum-mean-squared-error (LMMSE) as follows:

(22) $$\beta =\frac{\sigma_{diff}^{2}}{\sigma_{diff}^{2}+\sigma_{n}^{2}}$$

where $\sigma_{diff}^{2}$ is the sum of the local variance of the absolute frame difference from the center pixel to be denoised and the candidate motion vector position (19), which is determined by

(23) $$\sigma_{diff}^{2}=\frac{\sum_{k=1}^{4}\sigma_{y_{m,n,t-1,k}^{mc}}^{2}}{4}+\frac{\sum_{i=m-S}^{m+S}\sum_{j=n-S}^{n+S}{\left(|y_{m+i,n+i,t}-y_{m+i,n+j,t-1}|-\mu_{diff}\right)}^{2}}{n(S)}$$

(24) $$\mu_{diff}=\frac{\sum_{i=m-S}^{m+S}\sum_{j=n-S}^{n+S}\left|y_{m+i,n+i,t}-y_{m+i,n+j,t-1}\right|}{n(S)}$$

The motion adaptive detection value reflects motion changes in the local region of the CFA sequence and the candidate motion vectors from hypothetical intensity maps around the center pixel. A large $\sigma_{diff}^{2}$ implies that the correlation in the temporal domain between the center pixel and the result of motion-compensated temporal filtering is low when large scene changes and occlusions occur. In that case, the motion detection value β approaches one, which forces the weight of temporal filtered value ${\hat{x}}_{m,n,t-1}^{tf}$ to be small. The purely temporal filter shows limited denoising performance. Thus, the spatial filter must fully perform the denoising process. Therefore, the motion blurring that occurs with video denoising can be prevented. 

**Figure 8:**
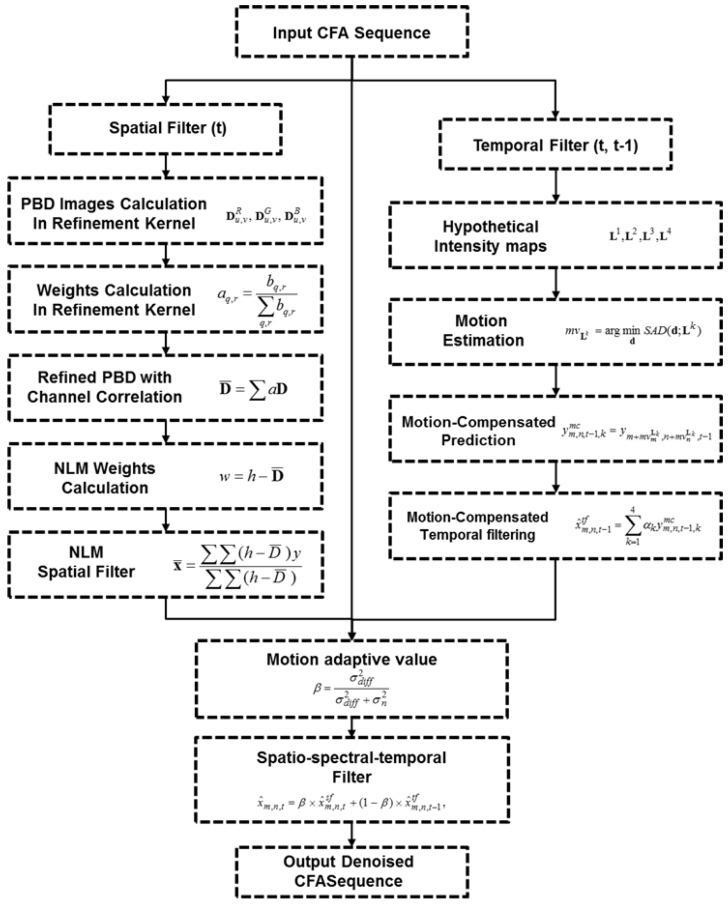
Overall flowchart of the proposed method.

The final denoising procedure is a combination of the spatial filter and temporal filter. This combination reduces the portion of spatial filtering, while retaining the advantages of the temporal filter, which does not produce oversmoothed denoising results. The motion detection value between the strength of the spatial filter and that of the temporal filter is optimally and adaptively controlled. In sequences with motions, the spatio-spectral-temporal denoising algorithm is performed mainly in the spatial domain. Additional temporal filtering procedures are conducted in the static sequences. Figure 8 illustrates the flowchart of the proposed method.

## 4. Experimental Results

We experimentally compared the numerical and visual performance of the proposed method with state-of-the-art denoising methods for a noisy CFA sequence, specifically first the single frame denoising [31] followed by demosaicing [15], first demosaicing [15] and then video denoising (VBM3D) [9], and then joint denoising and demosaicing for video CFA data [32]. The single frame denoising method [31] exploits characteristics of the human visual system and sensor noise statistics. In the demosaicing method [15], the LMMSE filtering for considering spatial and spectral correlations was utilized to reconstruct a full color image. The state-of-the-art grayscale video denoising method, VBM3D [9], was used next for the demosaicing method [15]. In [32], the denoising algorithm was used in the PCA-transformed domain to suppress the CFA image noise. Then, demosaicing was applied to the denoised CFA data.

**Figure 9:**
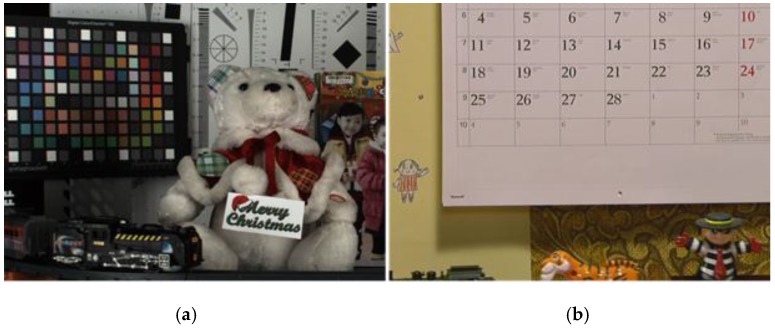
Scenes of two video sequence. (**a**) The *Moving train* sequence acquired by a CFA sensor; (**b**) The *Calendar* sequence.

The performance of the proposed method was tested with a real captured CFA sequence by using a Bayer pattern camera and a well-known common video sequence to simulate the CFA sequence, as shown in Figure 9. 

The original spatial resolution of each image was 1280 × 720 pixels. The original sequence shown in Figure 9a was captured by using CFA patterned camera. We then evaluated the denoising methods by adding Gaussian noise to the CFA sequence. The original sequence in Figure 9b is a full color sequence. The video data can be accessed at https://media.xiph.org/video/derf/. We down-sampled the sequence using the Bayer pattern and added Gaussian noise to evaluate the denoising methods. In this experiment, we attempted to remove the effect of demosaicing and evaluate the performance of the denoising method. In addition, gamma correction was applied under the same conditions for all the cases—original, noisy, and denoised sequences.

For color images, the image quality of the denoised and demosaiced results depend on the demosaicing methods. The demosaicing method used in [15] was adopted in [9] and [31], as well as in the proposed denoising method, whereby denoising and the demosaicing algorithm were separately conducted. Although denoising and demosaicing were performed together in [32], a similar method described in [15] was used in the internal joint process. 

**Figure 10:**
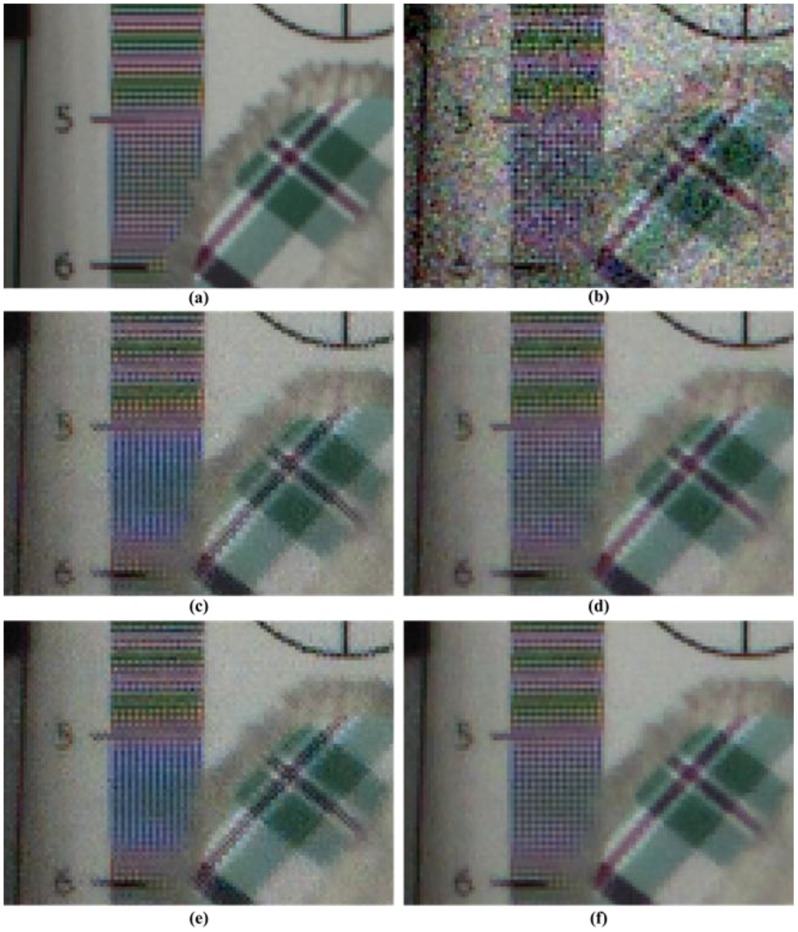
Partially magnified the *Moving train* sequence. (**a**) Original image; (**b**) Color demosaiced image obtained by [15] for noisy image with σ=10 of Bayer pattern; (**c**) Denoised image by method [31] with demosaicing of [15]; (**d**) Denoised image by method [9] with demosaicing of [15]; (**e**) Denoised image by method [32]; (**f**) Denoised image by proposed method with demosaicing of [15].

**Figure 11:**
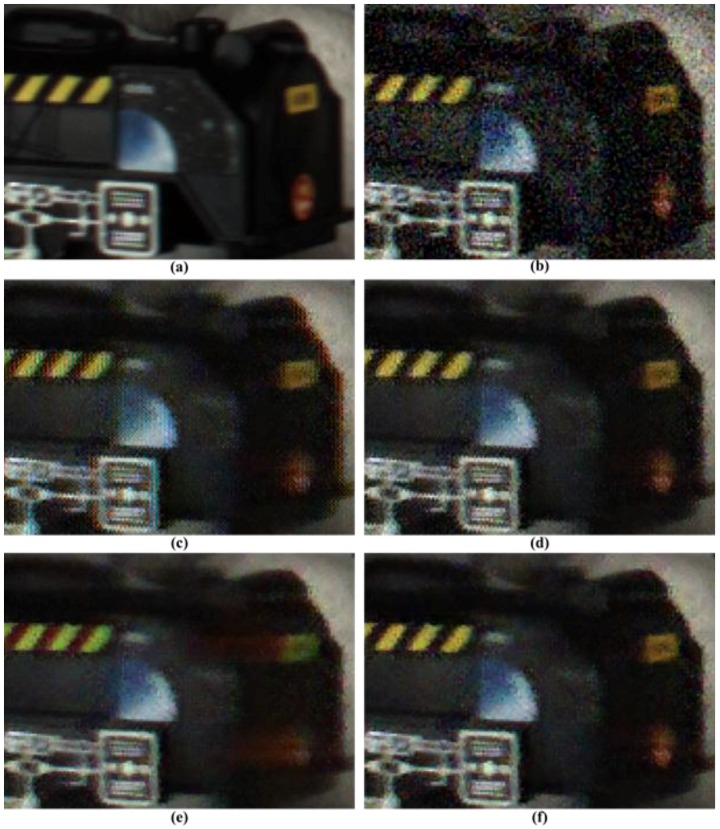
Partially magnified the *Moving train* sequence. (**a**) Original image; (**b**) Color demosaiced image obtained by [15] for noisy image with σ=10 of Bayer pattern; (**c**) Denoised image by method [31] with demosaicing of [15]; (**d**) Denoised image by method [9] with demosaicing of [15]; (**e**) Denoised image by method [32]; (**f**) Denoised image by proposed method with demosaicing of [15].

A visual comparison of the results is important for the algorithm evaluation. Figure 10 and Figure 11 illustrate a magnified view of some challenging parts of the restored “Moving Train” sequence. It is clearly seen that the proposed method significantly outperforms the conventional methods. As illustrated in Figure 10 and Figure 11, the visual results of the proposed method are better than those of the other methods. Although the method from [31] effectively removes noise and preserves edges or textures, color artifacts appear, especially in the characters, as shown in Figure 10c. The remaining noise appears in the results of Figure 10c and Figure 11c compared with the proposed method. The proposed method provides fewer color artifacts than the denoising method [31].

Although the VBM3D denoising method [9] demonstrates improved visual performance compared with the other methods, noise and ringing artifacts remain in the edge and detail regions, as shown in Figure 10d and Figure 11d. Because demosaicing process before denoising changes the statistical characteristics of the noise and spreads spatially. The method described in [32] has severe motion artifacts around the edges and details in the region of motion, as shown in Figure 11e. Because method [32] did not consider the inter-channel correlation in video denoising process using motion information. The high-frequency regions, which are difficult to denoise, including small details and textures, are preserved significantly better and the color artifacts are reduced in the proposed method, as shown in Figure 10f. Additionally, moving details are better preserved than those in the other methods, as shown in Figure 11f.

**Figure 12:**
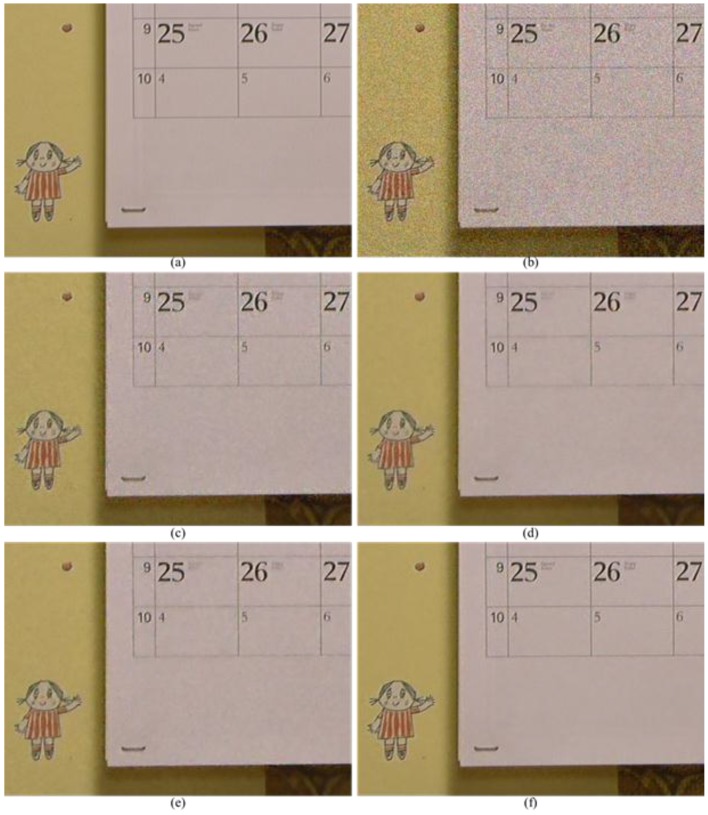
Partially magnified the *Calendar* sequence. (**a**) Original image; (**b**) Color demosaiced image obtained by [15] for noisy image with σ=10 of Bayer pattern; (**c**) Denoised image by method [31] with demosaicing of [15]; (**d**) Denoised image by method [9] with demosaicing of [15]; (**e**) Denoised image by method [32]; (**f**) Denoised image by proposed method with demosaicing of [15].

Figure 12 presents comparisons of the denoising results for relatively common video data, “Calendar”. Similar results were obtained in the simulation tests as in the captured set. The methods discussed in [31] demonstrate ringing artifacts around the edges and over-smoothed results in the detail regions, as shown in Figure 12c. Although the denoised results are clear and definite in [9] and [32], over-shooting and color artifacts appear around the strong edges, as shown in Figure 12d,e. In the proposed method in Figure 12f, the denoised results are more explicit and better than those in the other methods. It is evident that the proposed method provides significantly superior performance for the considerably noisy images that contain high-frequency regions that are difficult to restore.

**Figure 13:**
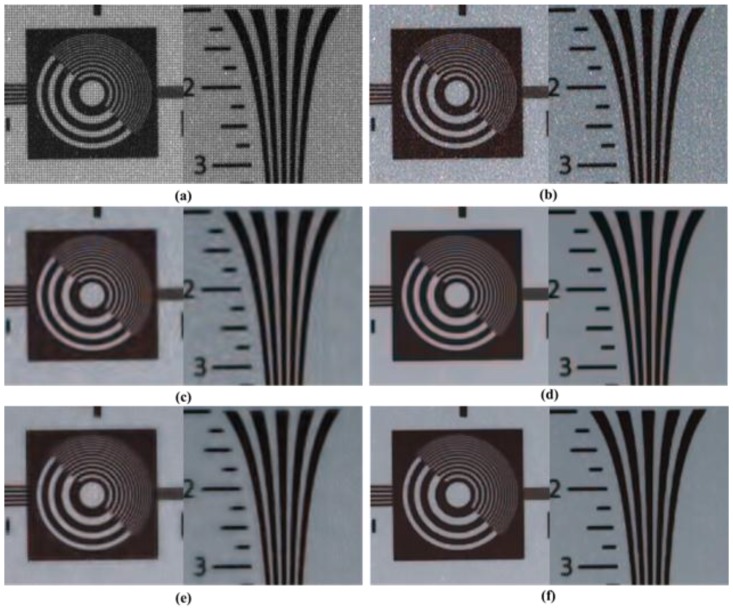
Partially magnified the real image. (**a**) Noisy image of Bayer pattern; (**b**) Color demosaiced image obtained by [15] for Bayer pattern; (**c**) Denoised image by method [31] with demosaicing of [15]; (**d**) Denoised image by method [9] with demosaicing of [15]; (**e**) Denoised image by method [32]; (**f**) Denoised image by proposed method with demosaicing of [15].

Figure 13 shows the denoising results obtained using different methods for a real static scene of original CFA image under noisy circumstances. Figure 13 is a difficult part in the processing of denoising and demosaicing. Similar results were obtained as in the simulation tests for the results of Figure 9. The methods discussed in [31] and [32] do not eliminate ringing artifacts around the edges and over-smoothed results in the detail regions. Although the denoised results are neat and definite for the method proposed in [9], false color and artifacts appear around the strong edges. Denoised results obtained using the proposed method are more definite and better than those obtained using the other methods. The proposed method also provides significantly superior performance for the considerably noisy images that contain high frequency regions, which are difficult to restore.

The numerical evaluation is presented in Table 1 and Table 2 for average peak signal to noise ratio (PSNR) and color peak signal to noise ratio (CPSNR) values. Table 1 and Table 2 display the average PSNR and CPSNR values of the algorithms at each noise level for the denoised results of the CFA sequence.

The color peak signal to noise ratio (CPSNR) is defined as:

(25) $$\mathrm{CPSNR}={10\log}_{10}\frac{255^{2}}{\mathrm{CMSE}},$$

where CMSE is represented as:

(26) $$\mathrm{CMSE}=\sum_{m=B+1}^{M-B}\sum_{m=B+1}^{N-B}\frac{{\left({\bar{x}}_{m,n}^{R}-x_{m,n}^{R}\right)}^{2}+{\left({\bar{x}}_{m,n}^{G}-x_{m,n}^{G}\right)}^{2}+{\left({\bar{x}}_{m,n}^{B}-x_{m,n}^{B}\right)}^{2}}{3(M-2B)(N-2B)},$$

where $x_{m,n}^{R}$, $x_{m,n}^{G}$, and $x_{m,n}^{B}$ are the true pixel values of the original color images for the R, G, and B channels, respectively, and ${\bar{x}}_{m,n}^{R}$, ${\bar{x}}_{m,n}^{G}$, and ${\bar{x}}_{m,n}^{B}$ are the estimated pixel values of the denoised images for the R, G, and B channels, respectively. M and N are the row and column sizes of the images, and B is the size of the border, which is not calculated for the CPSNR.

We evaluated the effectiveness of the proposed algorithm by using structural similarity (SSIM) measurements [34] for another representation of the performance. Various experimental setups were employed to examine the performance under the different noise levels and various motion scenarios. The SSIMs of the different scenes and noise levels were then calculated. The SSIM is defined as follows:

(27) $$SSIM=\frac{\left(2\mu_{x}\mu_{y}+C_{1})(2\sigma_{xy}+C_{2}\right)}{\left(\mu_{x}^{2}+\mu_{y}^{2}+C_{1})(\sigma_{x}^{2}+\sigma_{y}^{2}+C_{2}\right)},$$

where $\mu_{x}$ is the mean value of the denoised signal, $\mu_{y}$ is the mean value of the original signal, and ($\sigma_{x}$, $\sigma_{y}$) is the standard deviation of the signal. $C_{1}$ and $C_{2}$ are the variables used to stabilize the division with a weak denominator. The SSIM index is a decimal value between −1 and 1, and 1 is only reachable in case of two identical sets of data. Two different scenes in Figure 9 were chosen. For these different scenes, the average SSIMs at two noise levels were calculated, and the results are presented in Table 3 and Table 4. In general, our method outperformed other approaches in terms of average SSIM measurements. That is, the denoising result of the proposed method bears the most similarity to the original image.

In general, our method outperformed the other approaches in terms of the PSNR values. We observe that the proposed method produced superior results compared to the methods described in [31] and [32], which directly denoised the CFA image. The method used, VBM3D [9], which provides state-of-the-art results among the considered denoising techniques in the grayscale sequence, performed the second best. In summary, the proposed method outperformed the competition in terms of visual quality and numerical results. This is because the proposed method simultaneously and efficiently considers inter-channel and intra-channel correlations to suppress noise and preserve edges and fine details without color artifacts and motion artifacts.

In the general framework, the motion-compensated spatial-temporal filter is performed in the demosaiced full color sequence. A denoising filter in that framework is generally applied to the luminance domain. In the proposed framework, the denoising filter is applied to the CFA sequence before demosaicing. The proposed method can prevent color artifacts and the spread of noise during denoising process by considering spatio-tempo-spectral correlation. However, in the general framework, demosaicing performed in noisy CFA can cause changes in the statistical characteristics of the noise and degrades the spatial resolution. If the proposed method is implemented in a general framework, noise will be removed without considering inter-channel correlation. The spectral correlation in CFA sequence should be modified and applied to the denoising process in the general framework. 

## 5. Conclusions

In this paper, a spatio-spectral-temporal denoising filter considering the inter-channel correlation was proposed. The conventional PBD refinement process for monochromatic images is applied to the spatial filter to CFA images by analyzing the inter-channel correlation among the PBDs of the different channels. This is performed in a manner similar to the smooth assumption of the channel differences for demosaicing. A motion-compensated temporal filter based on MCP using inter-channel correlated motion estimation was additionally proposed. The denoised edges and details with enhanced resolution obtained by overcoming the degraded under-sampling contribute to the PBD refinement among the different channels. In the proposed method, moving details are well preserved without motion artifacts. Motion adaptive detection weight controls the results of the spatial filter and temporal filter according to the temporal correlations. The proposed method demonstrated good denoising performance and preserved the detailed motion regions during the denoising process without producing over-smoothness and artifacts when compared with the conventional denoising filters.

## 6. Future Work

Since the development of the Bayer pattern [16], various CFA patterns have been researched to minimize the degradation of resolution and enhance the sensitivity [19,20,21,22]. While many CFA patterns have been developed, Bayer pattern is still widely used for a single image sensor. The aliasing and grid effect in other CFA patterns become more prominent and varies with different lighting conditions [23]. Moreover, most existing demosaicing methods for the Bayer pattern cannot be applied to these patterns. To overcome these problems, many methods including demosaicing for other CFA patterns have also been researched recently [23,24]. The proposed algorithm is designed for Bayer pattern in a CFA single image sensor. The proposed framework can also be applied to other patterns. Future work will include the extension of the proposed method for other CFA patterns.

## Figures and Tables

**Table 1:** Average PSNR(dB) comparisons for the denoised and color interpolated results of *Moving train* sequence.

Noise level	[31] + [15]		[15] + [9]		[32]		Proposed + [15]	
	R PSNR	G PSNR	R PSNR	G PSNR	R PSNR	G PSNR	R PSNR	G PSNR
	B PSNR	CPSNR	B PSNR	CPSNR	B PSNR	CPSNR	B PSNR	CPSNR
**σ = 10**	30.76	31.53	31.13	31.91	30.37	31.02	**31.62**	**32.07**
	30.79	31.01	31.11	31.37	30.57	30.65	**31.75**	**31.84**
**σ = 20**	26.29	26.78	27.28	27.88	26.39	26.74	**27.94**	**28.37**
	26.50	26.52	27.45	27.53	26.63	26.58	**28.19**	**28.16**

**Table 2:** Average PSNR(dB) comparisons for the denoised and color interpolated results of *Calendar* sequence.

Noise level	[31] + [15]		[15] + [9]		[32]		Proposed + [15]	
	R PSNR	G PSNR	R PSNR	G PSNR	R PSNR	G PSNR	R PSNR	G PSNR
	B PSNR	CPSNR	B PSNR	CPSNR	B PSNR	CPSNR	B PSNR	CPSNR
**σ = 10**	30.64	31.22	31.39	32.06	30.57	30.90	**31.88**	**32.19**
	29.61	30.44	30.19	31.14	29.63	30.33	**30.68**	**31.54**
**σ = 20**	27.37	27.72	28.38	28.71	27.54	27.50	**28.50**	**28.89**
	26.76	27.26	27.64	28.22	26.84	27.28	**27.79**	**28.81**

**Table 3:** Average SSIM comparisons for the denoised and color interpolated results of *Moving train* sequence.

Noise level	[31] + [15]	[15] + [9]	[32]	Proposed + [15]
**σ = 10**	0.9138	0.9396	0.9124	**0.9495**
**σ = 20**	0.8845	0.8945	0.8788	**0.9098**

**Table 4:** Average SSIM comparisons for the denoised and color interpolated results of *Calendar* sequence.

Noise level	[31] + [15]	[15] + [9]	[32]	Proposed + [15]
**σ = 10**	0.9175	0.9268	0.9184	**0.9301**
**σ = 20**	0.8901	0.9001	0.8823	**0.9030**
